# Serum interleukin-40 increases in anti-AchR antibody-positive myasthenia gravis and correlates with disease activity

**DOI:** 10.3389/fneur.2025.1680530

**Published:** 2025-09-17

**Authors:** Kun Jia, Yingzhe Shao, Taofeng Tan, Xuan Liu, Qiuxia Zhang, Ning Zhao, Li Yang

**Affiliations:** ^1^Department of Neurology, Tianjin Huanhu Hospital, Tianjin, China; ^2^Department of Neurology, Tianjin Neurological Institute, Tianjin Medical University General Hospital, Tianjin, China; ^3^Department of Neurology, Tianjin Medical University General Hospital Airport Site, Tianjin, China

**Keywords:** myasthenia gravis, interleukin-40, biomaker, human, ELISA

## Abstract

**Background:**

Interleukin-40 (IL-40), as an immune regulatory factor discovered in recent years, mainly plays a role in B-cell-related immune responses and is involved in the pathological processes of various inflammatory diseases, autoimmune disorders, and infectious diseases. However, its role in myasthenia gravis (MG) has rarely been reported.

**Methods:**

We used enzyme-linked immunosorbent assay (ELISA) to measure the serum IL-40 levels in 58 MG patients and 55 healthy controls, and conducted a detailed analysis of the clinical data.

**Results:**

The serum IL-40 level in MG patients was significantly higher than that in healthy controls (*p* < 0.0001). After immunotherapy, the serum IL-40 level in MG patients significantly decreased (*p* < 0.0001). In MG, the IL-40 level of severe patients was significantly higher than that of mild patients (*p* < 0.0001). The ROC curve determined that the cut-off value for distinguishing IL-40 in MG from healthy controls was 15.63 pg/mL, with an AUC of 0.846 (95% CI: 0.773–0.919), 74.1% specificity, and 85.5% sensitivity.

**Conclusion:**

The serum IL-40 level in MG patients is elevated and is correlated with the severity of the disease. High levels of IL-40 may serve as a specific indicator for monitoring disease activity, which supports its potential as a non-invasive biomarker for disease monitoring.

## Introduction

Myasthenia gravis (MG) is a chronic autoimmune disorder characterized by fluctuating weakness and fatigue of skeletal muscles, with symptoms typically worsening after activity and improving with rest. The underlying pathophysiological mechanism involves the production of autoantibodies targeting key proteins at the neuromuscular junction (1). The most prevalent of these is the acetylcholine receptor (AChR) antibody, present in approximately 85% of patients (2). Other associated antibodies include those directed against lipoprotein-related protein 4 (LRP4), titin, ryanodine receptor, agrin, cortactin, and muscle-specific tyrosine kinase (MuSK) (3, 4). While these autoantibodies directly destroy the neuromuscular junction, inflammatory factors also play a crucial and multifaceted role in the initiation, progression, and exacerbation of the disease. These factors form a complex signaling network that facilitates communication between immune cells and tissue cells, thereby driving and amplifying the autoimmune response (5).

Interleukin-40 (IL-40) is a newly identified B-cell-associated cytokine encoded by the C17orf99 gene, with an approximate molecular weight of 27 kDa. It was first identified in 2017 and is highly expressed in mammalian fetal liver, bone marrow, and activated B cells, where it participates in the regulation of humoral immune responses and B-cell homeostasis (6). Recent studies have demonstrated that IL-40 plays a significant role in various inflammatory conditions, including rheumatoid arthritis, osteoarthritis, systemic sclerosis, and sepsis (7). However, its involvement in MG remains unexplored. In this study, we assessed serum IL-40 levels in patients with MG and analyzed their potential correlations with clinical parameters.

## Materials and methods

### Patients

We enrolled 58 MG patients who were treated at the Department of Neurology, Tianjin Medical University General Hospital between January 2020 and November 2023, and at the same time, 55 healthy controls (HCs) were also included in this study. The diagnosis of patients with MG is based on typical clinical manifestations and at least one of the following three criteria: (1) positive anti-AChR antibody detection in serum; (2) repetitive motor nerve stimulation (RNS) decrement of 10% or greater; and (3) a positive response to intramuscular neostigmine. The exclusion criteria were as follows: (1) age under 18 years and (2) a history of prior inflammatory, neoplastic, or other autoimmune diseases. HCs were recruited from the Health Care Center, and individuals with concomitant autoimmune diseases, infectious diseases, cardiovascular diseases, respiratory diseases, or renal diseases were excluded.

The study was approved by the Ethics Committee of Tianjin Medical University General Hospital, and all the participants provided written consent.

### Clinical data

Clinical data were extracted from the hospital’s electronic medical record system and included age, gender, anti-AChR antibody status, thymoma status, Myasthenia Gravis Foundation of America (MGFA) classification, and MG-ADL scores. Blood samples were collected from 58 MG patients prior to immunotherapy (intravenous immunoglobulin, plasma exchange or high-dose corticosteroids for 3–5 consecutive days). Moreover, additional samples were obtained during the remission phase from 22 of these patients. Concurrently, demographic data and venous blood samples were collected from 55 HCs. All blood specimens were collected from the elbow veins of the subjects (3–5 mL), transferred into serum separation tubes, centrifuged, and subsequently stored uniformly at −80 °C for batch analysis. Specimens from the acute phase were collected upon admission, while those from the remission phase were obtained after completion of immunotherapy.

### Measurement of serum IL-40 levels

Serum IL-40 levels were determined using human IL-40 enzyme-linked immunosorbent assay (ELISA) kit (Jianglai Biology, Shanghai, China). All procedures were conducted in strict adherence to the experimental protocol, and duplicate testing was carried out on both the standard and test samples.

### Statistical analyses

The normality of the data distribution was determined by Kolmogorov–Smirnov test. Serum IL-40 levels were compared between two groups using *t*-tests or the Mann–Whitney *U* test, gender was analyzed using the Fisher’s exact test. Correlations were analyzed using Spearman’s rank test. A Wilcoxon matched-pairs signed rank test was applied for the paired data. Data are expressed as mean ± standard deviation (SD). The area under the curve (AUC) in the receiver operating characteristic (ROC) analysis was used to evaluate the diagnostic efficacy of IL-40. The optimal cutoff values were determined by maximizing the Youden index (*J*), defined as *J* = Sensitivity + Specificity − 1. The AUC was interpreted according to the following criteria (8): 1.0 as perfect; 0.9–0.99 as excellent; 0.8–0.89 as good; 0.7–0.79 as fair; and ≤0.69 as poor or no value. *p* < 0.05 was considered statistically significant. Statistical analyses were performed using SPSS (version 27.0; IBM SPSS Statistics, IBM Corporation, Chicago, IL, United States) and GraphPad Prism software 10 (GraphPad Software Inc., San Diego, CA, United States).

## Results

### Clinical characteristics

The clinical features of the 58 MG patients and 55 HCs are shown in Table 1. All MG patients tested positive for anti-AChR antibodies. Given that most thymoma patients in this study received immunosuppressive therapy during specimen collection, individuals with concurrent thymoma were excluded. In this study, we defined mild MG patients as those who were classified as I or II by MGFA, and the rest of the patients were considered to be severe MG patients (9).

**Table 1:** Clinical characteristics of the study participants.

	MG	HC	*p*-value
Number	58	55	
Males/Females	29/29	28/27	0.923
Age, years	61.06 ± 14.99	60.93 ± 7.63	0.292
MG-ADL	5.38 ± 2.15	N/A	
MGFA classification (No. %)		N/A	
I	11 (19.0)		
II	29 (50)		
III	15 (25.9)		
IV	2 (3.4)		
V	1 (1.7)		

### Serum IL-40 levels

**Figure 1:**
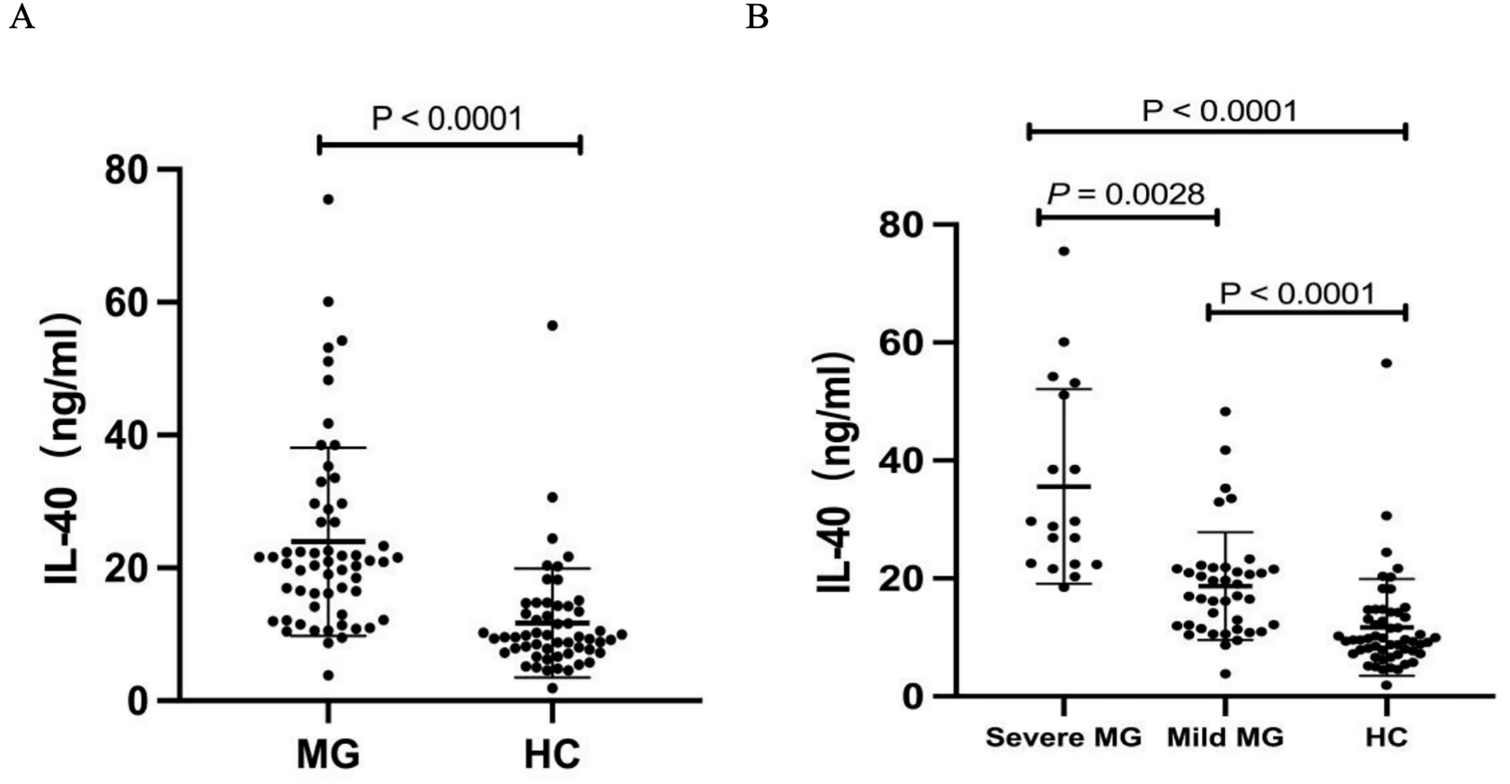
Serum interleukin 40 (IL-40) levels in patients with myasthenia gravis (MG). **(A)** IL-40 levels increased in patients with MG when compared with healthy controls (HCs). **(B)** IL-40 levels show a positive correlation with disease severity in patients with MG.

**Figure 2:**
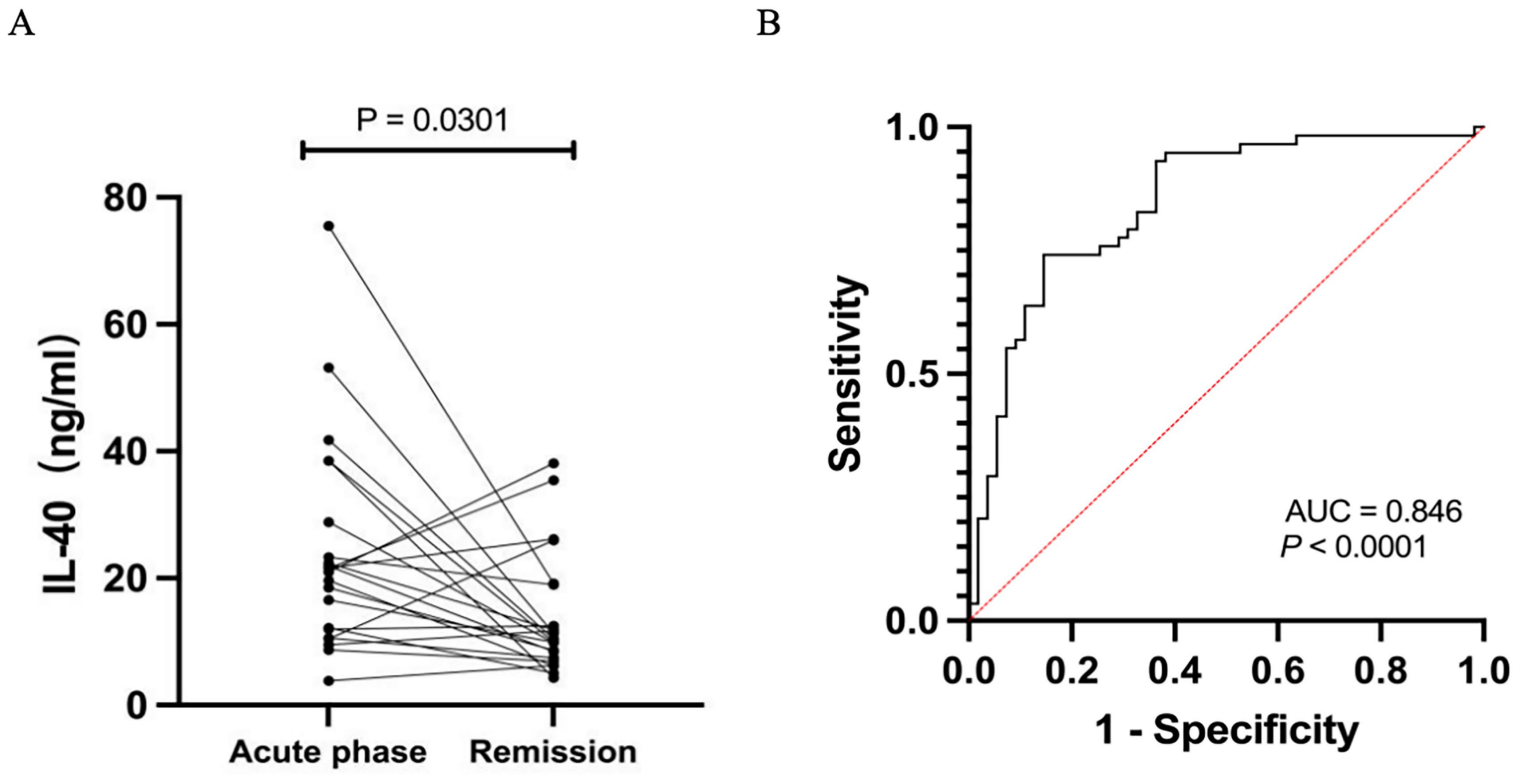
**(A)** Serum IL-40 level in MG patients during the remission phase, following immunotherapy, was significantly lower compared to that in patients during the acute phase. **(B)** The receiver operating characteristic (ROC) curve depicts the diagnostic performance of serum IL-40 in distinguishing patients with MG from HCs. The area under the curve (AUC) is 0.846 (95% CI: 0.773–0.919). The dashed red diagonal line represents the performance of random chance (AUC = 0.5).

Serum IL-40 levels in patients with MG were significantly higher than those in HCs (23.94 ± 14.14 vs. 11.69 ± 8.19 pg/mL, *p* < 0.0001) (Figure 1A). Moreover, these levels were markedly elevated in patients with severe MG compared to those with mild disease (35.60 ± 16.48 vs. 18.69 ± 9.13 pg/mL, *p* < 0.0001) (Figure 1B). Furthermore, our results demonstrated that serum IL-40 levels in MG patients significantly decreased following immunosuppressive treatment (24.08 ± 16.75 vs. 13.84 ± 9.54 pg/mL, *p* < 0.0001) (Figure 2A). However, no significant difference in serum IL-40 levels was observed between patients with ocular myasthenia gravis (OMG) and generalized myasthenia gravis (GMG) (*p* = 0.348). Additionally, no significant correlation was found between serum IL-40 levels and MG-ADL scores in patients with MG (*r* = 0.2324, *p* = 0.079). Receiver operating characteristic (ROC) analysis was conducted to assess the diagnostic performance of IL-40 in differentiating MG patients from HCs. The area under the curve (AUC) was 0.846 (95% CI: 0.773–0.919) for IL-40 in distinguishing MG patients from HCs (*p* < 0.0001) (Figure 2B). At the optimal cut-off value of 15.63 ng/mL, serum IL-40 levels demonstrated a sensitivity of 74.1% (95% CI: 61.6–83.7) and a specificity of 85.5% (95% CI: 73.8–92.4).

## Discussion

In MG, inflammatory factors serve as central mediators of the autoimmune response. They act synergistically to impair neuromuscular junction function through multiple mechanisms, including promoting B cell activation and pathogenic antibody production, exacerbating tissue damage and inflammatory cascades, and sustaining the chronic autoimmune cycle (10). IL-40 is a newly identified cytokine encoded by the C17orf99 gene. It exhibits no significant homology to known cytokine families, yet participates in diverse immune regulatory processes, inflammatory responses, and disease progression (7). In this study, we observed that serum IL-40 levels in patients with MG were significantly elevated compared to those in HCs, and these levels were positively correlated with disease severity. Furthermore, ROC curve analysis demonstrated that serum IL-40 exhibited high diagnostic specificity for distinguishing MG from HCs, with an AUC value of 0.846.

As a newly identified cytokine, research on IL-40 remains in its early stages, with the majority of studies concentrating on areas such as sepsis, autoimmune diseases, and fibrosis. IL-40 levels were significantly elevated in patients with sepsis upon admission, and were positively correlated with PCT, CRP, lactate and SOFA scores. Through single-cell RNA sequencing, it was discovered that IL-40 promotes the migration of S100A8/9hi neutrophils to the peritoneum and the formation of neutrophil extracellular traps (NETs), exacerbating cytokine storm and multiple organ failure (11). In rheumatoid arthritis, IL-40 is highly expressed in the synovial lining and infiltrating B cells, and is positively correlated with the levels of rheumatoid factor (RF-IgM) and anti-CCP antibodies (12). Furthermore, IL-40 is predominantly found in the superficial layer of cartilage in patients with osteoarthritis, where it activates chondrocytes to produce pro-inflammatory cytokines such as IL-6 and IL-8, as well as matrix-degrading enzymes including MMP-1, MMP-3, and MMP-13, thereby promoting cartilage degradation (13). However, the receptor for IL-40 and its downstream signaling pathways remain to be fully elucidated. IL-40 is predominantly secreted by activated B cells and has been shown to promote B cell proliferation, differentiation into plasma cells, and antibody class switching. Evidence from gene knockout studies indicates that the absence of IL-40 results in dysregulated B cell populations and impaired IgA secretion, suggesting that IL-40 plays a crucial role in maintaining B cell homeostasis (11). Given that B cells and NETs are key contributors to the pathogenesis of MG (10, 14), their interaction may give rise to a self-sustaining pathological cycle. Therefore, we propose that IL-40 may facilitate the progression of inflammatory diseases through a multi-cellular cooperative network involving B cells, neutrophils, and stromal cells, as well as multiple interlinked signaling pathways. Further investigation is required to validate this hypothesis.

It should be noted that this study has several limitations. First, as a cross-sectional study, it inherently carries a risk of bias. Second, the study was conducted at a single center with a relatively small sample size, which may affect the generalizability of the findings. Third, the exclusion of patients with other positive MG-related antibodies may introduce a degree of selection bias, potentially compromising the representativeness of the results. In future work, we aim to expand the sample size and further investigate the inflammatory mechanisms of IL-40.

## Conclusion

Our research has demonstrated that serum IL-40 levels are significantly elevated in patients with MG and are positively correlated with disease severity. Furthermore, IL-40 may serve as a promising biomarker for monitoring disease activity.

## Data Availability

The data that support the findings of this study are available from the corresponding author upon reasonable request.

## References

[1] Gilhus NE Verschuuren JJ. Myasthenia gravis: subgroup classification and therapeutic strategies. Lancet Neurol. (2015) 14:1023–36. doi: 10.1016/S1474-4422(15)00145-3, PMID: 26376969

[2] Mane-Damas M Schottler AK Marcuse F Molenaar PC Mohile T Hoeijmakers JGJ. Myasthenia gravis with antibodies against the AChR, current knowledge on pathophysiology and an update on treatment strategies with special focus on targeting plasma cells. Autoimmun Rev. (2025) 24:103875. doi: 10.1016/j.autrev.2025.103875, PMID: 40651620

[3] Yan M Xing G Xiong W Mei L. Agrin and LRP4 antibodies as new biomarkers of myasthenia gravis. Ann N Y Acad Sci. (2018) 1413:126–35. doi: 10.1111/nyas.13573, PMID: 29377176

[4] Illa I Cortes-Vicente E Martinez MA Gallardo E. Diagnostic utility of cortactin antibodies in myasthenia gravis. Ann N Y Acad Sci. (2018) 1412:90–4. doi: 10.1111/nyas.13502, PMID: 29068555

[5] Shushtari A Ashayeri H Salmannezhad A Seyedmirzaei H Rezaei N. Pro-inflammatory cytokines in myasthenia gravis: a systematic review and meta-analysis. Neurol Sci. (2025). doi: 10.1007/s10072-025-08218-3, PMID: 40347402

[6] Catalan-Dibene J Vazquez MI Luu VP Nuccio S Karimzadeh A Kastenschmidt JM. Identification of IL-40, a novel B cell-associated cytokine. J Immunol. (2017) 199:3326–35. doi: 10.4049/jimmunol.1700534, PMID: 28978694 PMC5667921

[7] Dabbagh-Gorjani F. A comprehensive review on the role of Interleukin-40 as a biomarker for diagnosing inflammatory diseases. Autoimmune Dis. (2024) 2024:3968767. doi: 10.1155/2024/3968767, PMID: 38464677 PMC10923619

[8] Carter JV Pan J Rai SN Galandiuk S. ROC-ing along: evaluation and interpretation of receiver operating characteristic curves. Surgery. (2016) 159:1638–45. doi: 10.1016/j.surg.2015.12.029, PMID: 26962006

[9] Zhang Q Li Y Jiang S Zhang L Yi M Wang J. Increased serum IL-36gamma levels are associated with disease severity in myasthenia gravis patients. BMC Neurol. (2020) 20:307. doi: 10.1186/s12883-020-01885-z 32814555 PMC7436949

[10] Bayer AC Nowak RJ O’Connor KC. Contribution of cellular immune dysregulation to myasthenia gravis pathology. Int Rev Neurobiol. (2025) 182:43–66. doi: 10.1016/bs.irn.2025.04.035 40675740

[11] Cai S Li X Zhang C Jiang Y Liu Y He Z. Inhibition of Interleukin-40 prevents multi-organ damage during sepsis by blocking NETosis. Crit Care. (2025) 29:29. doi: 10.1186/s13054-025-05257-2, PMID: 39819454 PMC11740647

[12] Navratilova A Becvar V Hulejova H Tomcik M Stolova L Mann H. New pro-inflammatory cytokine IL-40 is produced by activated neutrophils and plays a role in the early stages of seropositive rheumatoid arthritis. RMD Open. (2023) 9:e002894. doi: 10.1136/rmdopen-2022-002894, PMID: 37208028 PMC10201233

[13] Cerezo LA Navratilova A Kuklova M Prokopcova A Baloun J Kropackova T. IL-40 is up-regulated in the synovial fluid and cartilage of osteoarthritis patients and contributes to the alteration of chondrocytes phenotype *in vitro*. Arthritis Res Ther. (2024) 26:146. doi: 10.1186/s13075-024-03372-z, PMID: 39080724 PMC11289996

[14] Zhang S Wen Q Su S Wang Y Wang J Xie N. Peripheral immune profiling highlights a dynamic role of low-density granulocytes in myasthenia gravis. J Autoimmun. (2025) 152:103395. doi: 10.1016/j.jaut.2025.103395, PMID: 40043622

